# Temporal stability in species richness but reordering in species abundances within avian assemblages of a tropical Andes conservation hot spot

**DOI:** 10.1111/btp.13016

**Published:** 2021-10-05

**Authors:** Boris A. Tinoco, Steven C. Latta, Pedro X. Astudillo, Andrea Nieto, Catherine H. Graham

**Affiliations:** ^1^ Escuela de Biología Universidad del Azuay Cuenca Ecuador; ^2^ National Aviary Allegheny Commons West Pittsburgh Pennsylvania USA; ^3^ Swiss Federal Research Institute WSL Birmensdorf, Zurich Switzerland

**Keywords:** Ecuador, global change, monitoring, montane forest, protected areas, reordering, turnover

## Abstract

As the pace of environmental change increases, there is an urgent need for quantitative data revealing the temporal dynamics of local communities in tropical areas. Here, we quantify the stability of avian assemblages in the highly threatened, but poorly studied, Andean biodiversity hot spot. We evaluated the temporal variation in species richness and community composition of local bird assemblages in three habitat types (native forest, introduced forest, native shrub) using a unique, relatively long‐term data series from Cajas National Park and Mazán Reserve in the southern Andes of Ecuador. We sampled birds with mist nets using a standardized protocol over 11 years, from 2006 to 2016. Species richness remained stable over time across habitats, but community composition changed in the native forest. In particular, we observed taxonomic reordering in the native forest, in which the evenness in the distribution of abundances of taxa decreased over time. This finding is consistent with other studies where species richness remained constant over time while community composition changed. Our study highlights the value of long‐term studies in the tropical Andes as we show that species composition of birds in a montane forest is changing, consistent with global trends in biodiversity change.

## Graphical abstract

We evaluated the temporal variation in species richness and community composition of local bird assemblages over 11 years in Cajas National Park and Mazán Reserve in the Southern Andes of Ecuador. Species richness remained stabled over time, but we observed taxonomic reordering in the undisturbed native forest, in which the evenness in the distribution of abundances of taxa decreased over time. This trend, resulting in increased dominance of some species, while others became rare in the native forest, is of conservation concern because it indicates that biodiversity could be changing over time even in the absence of local disturbances.

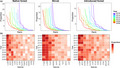

## Resumen

La tasa acelerada del incremento en el cambio ambiental crea la necesidad urgente de datos cuantitativos que expongan la dinámica temporal de comunidades locales en áreas tropicales. En este manuscrito, cuantificamos la estabilidad de ensambles de aves un área altamente amenazada pero poco estudiada, el hot‐spot de biodiversidad Andino. Evaluamos la variación temporal en riqueza y composición del ensamble de aves local en tres tipos de hábitat (bosque nativo, bosque introducido, matorrales nativos) usando una serie de datos de largo plazo relativo, obtenidos en el Parque Nacional Cajas y la reserva Mazan, en los Andes del sur del Ecuador. Las aves se estudiaron con redes de neblina por medio de protocolos estandarizados por 11 años, desde 1996 al 2016. La riqueza de especies permaneció estable en el tiempo en todos los hábitats, pero la composición de especies cambió en el bosque nativo. En particular, observamos un reorden taxonómico en el bosque nativo, en el cual existió una diminución en la equitatividad de las distribuciones de las especies en el tiempo. Este resultado es consistente con otros estudios que encuentran que la riqueza de especies permanece constante en el tiempo mientras que la composición de la comunidad cambia. Nuestro trabajo resalta el valor de los estudios de largo tiempo en los Andes tropicales al demostrar que la composición de aves está cambiando, y concuerda con los patrones globales de cambio de la biodiversidad.

## 1 INTRODUCTION

The increasing pace of environmental change has resulted in a rapid decline of populations, range shifts, and, in some cases, extinctions over the last century (Barnosky et al., 2011; Rosenberg et al., 2019). However, at the local scale species richness, somewhat paradoxically, is often stable over time (Blowes et al., 2019; Dornelas et al., 2014; Hillebrand et al., 2018). Local communities are part of metacommunities (Leibold et al., 2004), and as such, changes in species abundance or extinctions can be compensated for by immigration of individuals from the regional pool (Nielsen et al., 2019). As a result, species richness in local communities might be temporally stable (Magurran & Henderson, 2018), while the composition and abundance of species might be dynamic (Blowes et al., 2019; Larsen et al., 2018). Unfortunately, in many tropical biodiversity hot spots we lack temporal series of biodiversity data (Dornelas et al., 2018; Newbold et al., 2020), hindering our ability to quantify the patterns of variation in species richness and composition needed to identify generalities in the responses of biodiversity to global change.

**FIGURE 1:**
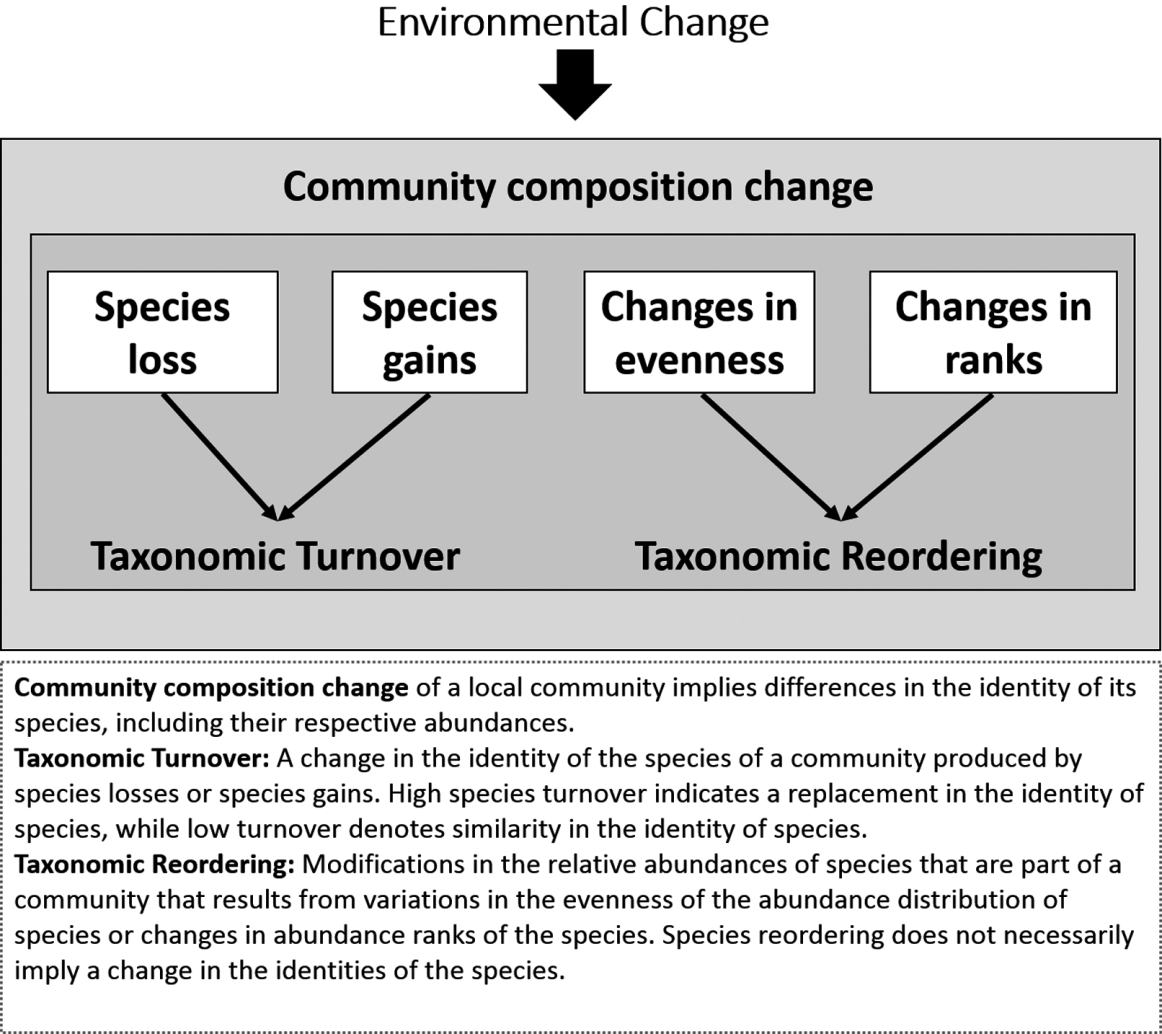
Schematic diagram of potential community responses to the effects of environmental change

Drivers of environmental change, such as climate and land‐use change, can influence temporal variation in local communities (i.e., species interacting in a given place during a given time period), where changes are generally quantified in terms of species richness and community composition (Magurran & Henderson, 2010; Newbold et al., 2020). A change in species richness simply results from the addition or reduction in the number of species. A change in community composition can be a consequence of taxonomic turnover, where the identity of co‐occurring species change, and/or taxonomic reordering, where the identity of the species persists but their relative abundances change (Avolio et al., 2015; Collins et al., 2020; Dornelas et al., 2013) (Figure 1). Taxonomic turnover results from the immigration of new species into a community, or from local extinctions (Buckley & Jetz, 2008; Shurin, 2007). Taxonomic reordering occurs when the evenness in the distribution of species abundance in a community changes (Heard et al., 2012; Song et al., 2019), or when the pattern of rank abundances of species changes (Collins et al., 2020; Jones et al., 2017) (Figure 1). Understanding the temporal change in local communities requires unraveling the influence of richness, turnover, and reordering in the dynamics of communities (Avolio et al., 2015).

The few long‐term studies of changes in biological communities carried out in the tropical region generally agree with the world‐wide pattern of biodiversity change (Blowes et al., 2019; but see Beaudrot et al., 2016). For instance, a reordering of tree species over 35 years was detected in a network of study plots in the Amazonia forest lowlands (Esquivel‐Muelbert et al., 2019). Dung beetle communities in the lowlands of Costa Rica, studied over a period of 30 years, presented a decline in species richness and taxonomic reordering (Escobar et al., 2008). A 20‐year decline in the species richness of mammals was detected in tropical Queensland, Australia (Laurance et al., 2008). For birds, a 13‐year study in the Amazonia lowlands found taxonomic reordering (Blake & Loiselle, 2016). However, long‐term studies in tropical ecosystems are still scarce, in particular in the highly threatened, but poorly studied tropical Andes (Blowes et al., 2019).

Protected areas in the tropical region play a pivotal role in the conservation of biodiversity (Bruner et al., 2001; Naughton‐Treves et al., 2005), but their effectiveness has been challenged (Laurance et al., 2012; Malhi et al., 2014). The biodiversity that such areas seek to conserve might be governed by metapopulation and metacommunity processes operating at scales larger than the protected area itself (Hansen & DeFries, 2007; Laurance et al., 2012). Reserves are embedded within a larger ecosystem, and they are not isolated from the effects of global climate or regional land‐use change (Hansen & DeFries, 2007; Maiorano et al., 2008); as a result, there is a global concern about changes in taxonomic richness and community composition within protected areas (Ponce‐Reyes et al., 2012; Velásquez‐Tibatá et al., 2013).

Cajas National Park and the nearby Mazán reserve, located in southern Ecuador, harbor a representative avifauna of high‐elevation tropical Andean ecosystems and have been recognized as an important bird conservation area (Astudillo et al., 2015; Freile & Santander, 2005). Previous work in this area reported a decline in bird species richness in native and introduced forest habitats of the Mazán reserve between 1994 and 2007 (Latta et al., 2011). However, these results were based on a comparison between two points in time (1994–1995 and 2006–2007), covering a sampling interval of 12 years; nonetheless, communities can have heterogeneous changes over time, and data derived from continuous time series are better suited for revealing the processes underlying community dynamics (Reinke et al., 2019; Stouffer et al., 2021; White, 2019).

In this study, we explored avian diversity change over a period of 11 years in Cajas National Park and Mazán reserve, using data that have been collected continuously from 2006 to 2016 in three habitats: native forest, introduced forest (i.e., forests planted with exotic trees), and native shrub. Specifically, we analyzed changes in local communities to ask the following three questions: (1) Is taxonomic richness changing over time? Given the previous evidence of decline in richness in the study area (Latta et al., 2011), we expect a decline in richness across all habitats; (2) Is species composition changing over time? We expect a temporal decline in composition similarity across different habitats, corresponding to the global trend of local community composition change over time (Blowes et al., 2019; Dornelas et al., 2014); (3) Is the change in species composition a result of turnover and/or the reordering of species (i.e., changes in abundances)? Given earlier results from Latta et al. (2011), we expect change will be driven by both reordering and turnover. Our moderately long‐term study offers an opportunity to reveal how communities are responding to the global environmental change in a biodiversity hot spot.

## 2 METHODS

### 2.1 Study area and study design

**FIGURE 2:**
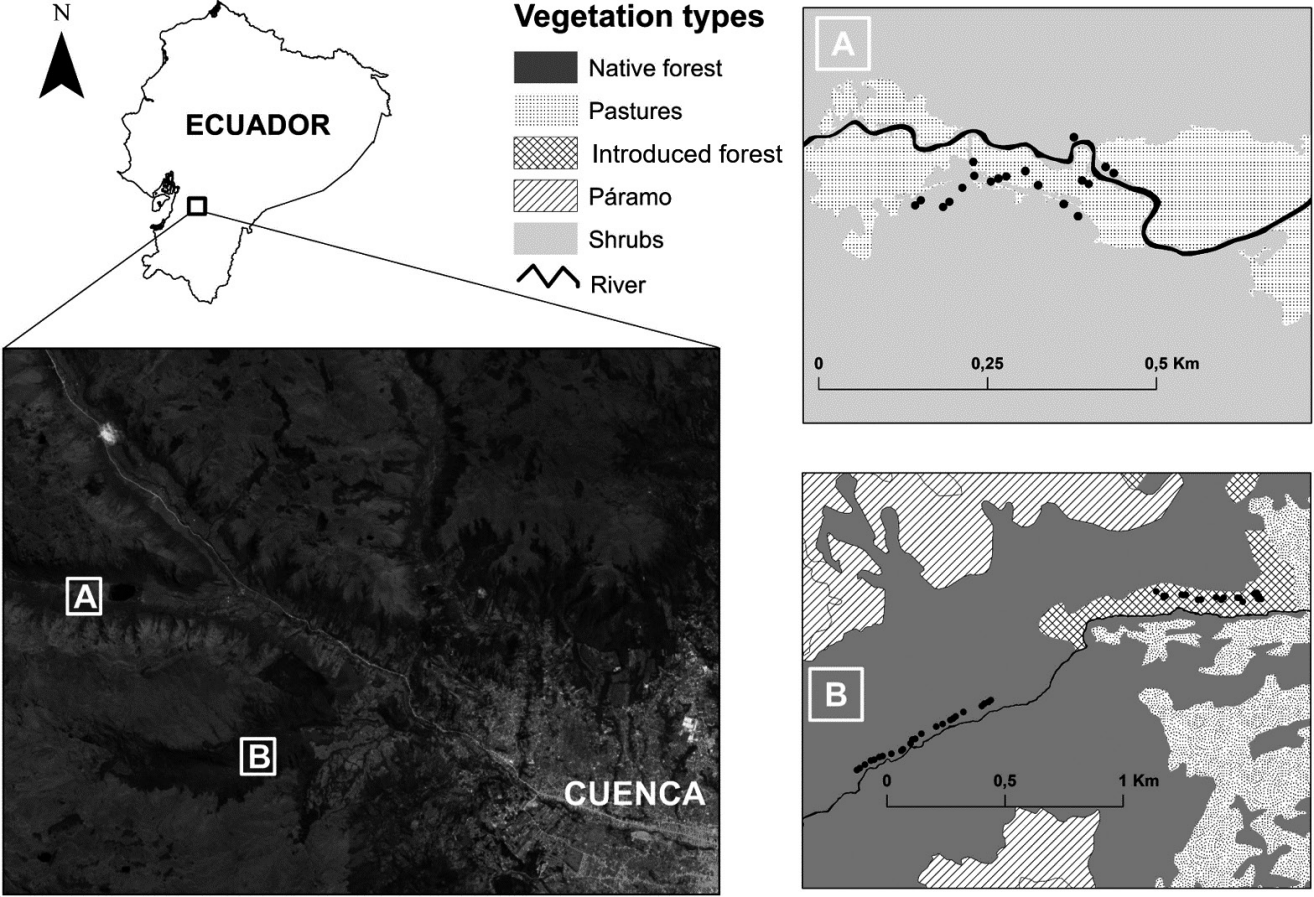
Map of the study area (Google, 2019) showing the location of Llaviuco valley (A) in Cajas National park and Mazán reserve (B). Black dots shown in the inlets A and B represent the location of the mist nets in each sampling station

This study was conducted in Cajas National Park and the adjacent Mazán private reserve in the high Andes of Azuay province, Ecuador (Figure 2). These areas are of global importance for biodiversity conservation, have been designated an Important Bird Area (IBA) (Freile & Santander, 2005), and are part of the “Macizo del Cajas” International Biosphere Reserve (UNESCO). The study area receives 1200–1500 mm of rain with a main rainy season from January to June, a dry season from July to September, and a secondary rainy season from October to December (Celleri et al., 2007). Mean monthly temperatures range from 5 to 12°C, but temperature variation within a day can range from 0 to 20°C (Celleri et al., 2007).

Mazán reserve has 2700 ha (02.870° S, 79.120° W) of mostly native montane forest, with páramo grasslands occupying the upper ridges of the valley (Figure 2). Selective logging occurred in Mazán >30 years ago, but today it is under strict protection that permits only limited scientific activity. In Cajas National Park, we worked in the U‐shaped Llaviuco valley (02.840 S, 79.160° W) at the eastern border of the park. Recreational activities (trekking) are common in this valley, but are limited to selected trails (Figure 2). Most of the forest in this valley was cleared for cattle ranching, but since its incorporation into Cajas National Park in 1996 and the removal of the cattle, vegetation has naturally re‐established.

Within Mazán and Llaviuco, we placed three sampling stations in areas with different habitat types: native montane forest (hereafter native forest), mixed stands of mature *Eucalyptus* sp. and *Pinus patula* with a native understory (hereafter introduced forest), and early successional vegetation (hereafter native shrub) (Figure 2). The native forest was located in Mazán at an elevation of 3200 m. a.s.l. Common tree species around the sampling station included *Hedyosmum cumbalense*, *Symplocos quitensis*, and *Myrcianthes* sp., with an understory mainly composed of *Miconia bracteolata*, *Viburnum triphyllum*, and *Oreopanax avicenniifolius*. The canopy reached 10–15 m. The introduced forest was also located in Mazán at 3100 m. a.s.l. The canopy here varied between 15 and 20 m, and species common in the understory included *Rubus* sp. and *Baccharis* sp. The native shrub was located in Llaviuco Valley at an elevation of 3150 m. a.s.l. and is successional stage of native montane forest. Common shrub species were *Barnadesia arborea*, *Brachyotum* sp., *Rubus floribundus*, and *Salvia corrugata*. The canopy was low (<2–3 m), with isolated taller trees mainly along small creeks that crossed our sampling area. There has been little change in the structure of the vegetation in all habitat types over the period of this study (Tinoco et al., 2019).

### 2.2 Sampling protocol

At each sampling station, we captured birds by placing 20 mist nets (12 m × 32 mm mesh) along edges of narrow trails (1 m approximately) in fixed locations for two consecutive days (Figure 2). Nets were open from dawn to 17:00 of day 1 and dawn to 11:00 of day 2. All birds were uniquely banded with a numbered aluminum metal band to identify recaptured individuals. Birds were sampled three times annually from 2006 to 2016 at each sampling station. To cover the climatic seasonality of the study area, we sampled once in the main wet season (within April 1–May 15), once in the dry season (within July 15–August 15), and once in the secondary rainy season (within November 9–December 18).

It is suspected that birds may sometimes learn the locations of nets over time and avoid being captured (Karr, 1981); however, avoidance of nets by previously captured birds likely lasts for only a few days (Ballard et al., 2004; Nur & Geupel, 1993). Therefore, we limited net avoidance biases by sampling only during two continuous days, with a minimum of 60 days between samplings periods.

The total number of sampled hours varied somewhat among sampling periods because we did not operate nets during rainy conditions; therefore, to consider variation in the sampling effort during each sampling period, both species richness and abundance were expressed as number of species or individuals captured in 100 mist net hours, where one mist net opened for 1 hr = 1 mist net hour.

### 2.3 Data analysis

We obtained annual taxonomic richness and abundance based on the three field samples collected each year; we used this as our unit for all analyses. Annual species richness was obtained by calculating the cumulative species richness in 100 mist net hours per year, and annual abundance for each species was estimated as its average abundance in 100 mist net hours among the three sampling periods per year. We used band numbers to avoid double‐counting birds within the sampling periods.

### 2.4 Across‐time comparisons

To determine whether species richness changed over time, we used linear models, with a normal distribution of residuals, for each habitat, with observed annual taxonomic richness as the response variable and time as the predictor variable. Species richness can be sensitive to variation in the detectability of species among samples (Boulinier et al., 1998); thus, we calculated detectability in species richness for each year in each habitat following Kéry and Schmid (2005), with the formula *D* = *C*/*N*, where *C* is the annual richness and *N* represents the potential richness. Potential richness (*N*) was estimated with a Jackknife estimator, obtained by using observed species richness of each of the three sampling periods within each year (Kéry & Schmid, 2005; Walther & Moore, 2005). Jackknife is a nonparametric richness estimator, based on a resampling technique that accounts for variation in detectability by leaving out one sample at a time and estimating averages over the rest of samples (Heltshe & Forrester, 1983). We explored if there was temporal variation in detectability in species richness over time with a linear regression.

We calculated the temporal rate of change in community composition in each habitat using time‐lag analysis (Collins et al., 2000). In time‐lag analysis, a measure of community dissimilarity among sampling periods at increasing time lags is calculated. For the eleven years of study, we obtained ten one‐year time lags (year 1 vs year 2, year 2 vs year 3, … year 10 vs year 11), nine two‐year time lags (year 1 vs year 3, year 2 vs year 4, … year 9 vs year 11), through one ten‐year time lag (year 1 vs year 11). Then, a linear regression between dissimilarity and time lags was used to evaluate whether there is directional change in community composition based on the slope of the regression (Collins et al., 2000). A matrix of species abundances recorded during each year was constructed for each habitat, from which we calculated dissimilarity in community composition among years using Euclidean distance. Euclidean distance was used because it incorporates information on joint absences between samples, and has clear geometric properties (Anderson et al., 2011; Collins et al., 2000).

Rare species can have a strong influence on dissimilarity measures, and those species could have been under‐sampled in our field method. We, therefore, repeated the time‐lag analysis including only dominant species, defined as the minimum number of species that together represent 50% of all individuals that are part of the assemblage of interest, which in our case was the annual assemblage each year for each habitat. That percentage (50%) has been previously shown to follow abrupt changes in the abundance curve of species found in Amazonian communities (Steege et al., 2013).

We further assessed if changes in species composition were the result of taxonomic turnover or taxonomic reordering. To explore the role of taxonomic turnover, we performed a time‐lag analysis including only species presence or absence in the dissimilarity distance calculations. If taxonomic turnover among temporal samples is important in a community, a significant rate of change over time should be detected (Jones et al., 2017). Two aspects of taxonomic reordering were evaluated: evenness and rank abundance shifts. To test for changes in evenness over time, we created linear regression models using the annual Pielou's index of evenness as a response variable and time as the predictor variable. Pielou's evenness captures the amount of evenness of a community relative to the maximum evenness possible for a given richness (equal abundance for all the species in the community) (Tuomisto, 2012). Rank abundance shifts were evaluated by measuring mean rank change in species across time (Avolio et al., 2019). Mean rank change calculates the degree of species reordering over time by comparing the amount of change in species ranks between continuous time periods (Avolio et al., 2019).

Lastly, to visualize changes in the communities over time we generated annual rank abundance distribution plots (Matthews & Whittaker, 2015), which depict different structural properties of assemblages such as changes in richness, evenness, and rank abundances (Matthews & Whittaker, 2015). To recognize the identity of the dominant species in each assemblage and to explore changes in species rank abundances over time, we used heat maps depicting the proportional abundance of dominant species.

The performance of all of the linear models described above was validated by visually exploring the distribution of residuals and homogeneity of variances; in all cases, the distribution of residuals indicated normality, and plots of residuals against fitted values reveal homogeneity of variances. Autocorrelation function plots (ACF) were used to check for potential temporal autocorrelations, and they did not indicate temporal autocorrelation of residuals. All of the analyses were performed using software R (R Development Core Team, 2020).

## 3 RESULTS

Overall, we captured 68 species across all habitats, with 50 species occurring in native forest, 57 species in native shrubs, and 49 species in the introduced forest (Table 1). Mean annual richness of species, expressed in 100 mist net hours, was 3.11 ± 0.44 in native forest, 4.99 ± 0.96 in native shrubs, and 3.62 ± 0.44 in the introduced forest. Some species were frequently caught in all habitats during the study period, including: *Coeligena iris*, *Heliangelus viola*, *Metallura tyrianthina*, *Scytalopus latrans*, *Myioborus melanocephalus*, *Diglossa cyanea*, *and D*. *humeralis* (Table 1).

**TABLE 1:** List of bird species captured with mist nets in native forest, native shrubs, and exotic forest habitats in Cajas National Park and Mazán reserve, southern highlands of Ecuador

Scientific name	English name	Species code	Habitat		
			Native Forest	Native Shrub	Exotic Forest
*Glaucidium jardinii*	Andean Pygmy‐Owl	GLJA	2	4	2
*Aglaeactis cupripennis*	Shining Sunbeam	AGCU	0	6	2
*Chaetocercus mulsant*	White‐bellied Woodstar	CHMU	0	2	0
*Coeligena iris*	Rainbow Starfrontlet	COIR	11	11	11
*Colibri coruscans*	Sparkling Violetear	COCO	0	4	1
*Ensifera ensifera*	Sword‐billed Hummingbird	ENEN	1	1	0
*Eriocnemis luciani*	Sapphire‐vented Puffleg	ERLU	9	11	11
*Eriocnemis vestita*	Glowing Puffleg	ERVE	3	9	1
*Heliangelus viola*	Purple‐throated Sunangel	HEVI	11	10	11
*Lafresnaya lafresnayi*	Mountain Velvetbreast	LALA	11	10	9
*Lesbia nuna*	Green‐tailed Trainbearer	LENU	1	5	4
*Lesbia victoriae*	Black‐tailed Trainbearer	LEVI	0	4	0
*Metallura baroni*	Violet‐throated Metaltail	MEBA	1	11	1
*Metallura tyrianthina*	Tyrian Metaltail	METY	11	11	11
*Pterophanes cyanopterus*	Great Sapphirewing	PTCY	2	9	0
*Trogon personatus*	Masked Trogon	TRPE	4	0	0
*Veniliornis nigriceps*	Bar‐bellied Woodpecker	VENI	0	2	0
*Colaptes rivolii*	Crimson‐mantled Woodpecker	PIRI	0	0	3
*Hellmayrea gularis*	White‐browed Spinetail	HEGU	8	7	11
*Margarornis squamiger*	Pearled Treerunner	MASQ	9	10	3
*Cranioleuca antisiensis*	Line‐cheeked Spinetail	CRAN	0	4	8
*Pseudocolaptes boissonneautii*	Streaked Tuftedcheek	PSBO	3	2	3
*Synallaxis azarae*	Azara's Spinetail	SYAZ	9	11	10
*Thripadectes flammulatus*	Flammulated Treehunter	THFL	7	0	4
*Grallaria quitensis*	Tawny Antpitta	GRQU	1	1	0
*Grallaria ruficapilla*	Chestnut‐crowned Antpitta	GRRF	2	0	2
*Grallaria rufula*	Rufous Antpitta	GRRU	9	7	11
*Grallaria squamigera*	Undulated Antpitta	GRSQ	2	0	2
*Scytalopus latrans*	Blackish Tapaculo	SCLA	10	11	11
*Anairetes parulus*	Tufted Tit‐Tyrant	ANPA	0	11	2
*Uromyias agilis*	Agile Tit‐Tyrant	URAG	1	0	1
*Elaenia albiceps*	White‐crested Elaenia	ELAL	2	3	0
*Mecocerculus leucophrys*	White‐throated Tyrannulet	MELE	0	6	0
*Mecocerculus stictopterus*	White‐banded Tyrannulet	MEST	4	3	0
*Myiotheretes fumigatus*	Smoky Bush‐Tyrant	MYFU	2	0	0
*Ochthoeca cinnamomeiventris*	Slaty‐backed Chat‐Tyrant	OCCI	3	0	4
*Ochthoeca frontalis*	Crowned Chat‐Tyrant	OCFR	10	10	9
*Ochthoeca fumicolor*	Brown‐backed Chat‐Tyrant	OCFU	1	3	0
*Ochthoeca rufipectoralis*	Rufous‐breasted Chat‐Tyrant	OCRU	2	1	0
*Phyllomyias uropygialis*	Tawny‐rumped Tyrannulet	PHUR	6	5	1
*Pyrrhomyias cinnamomeus*	Cinnamon Flycatcher	PYCI	0	0	3
*Cyanolyca turcosa*	Turquoise Jay	CYTU	2	1	4
*Turdus chiguanco*	Chiguanco Thrush	TUCH	2	0	0
*Turdus fuscater*	Great Thrush	TUFU	3	5	6
*Cistothorus platensis*	Sedge Wren	CIPL	0	11	1
*Troglodytes solstitialis*	Mountain Wren	TRSO	8	9	9
*Myioborus melanocephalus*	Spectacled Redstart	MYME	11	11	11
*Myioborus miniatus*	Slate‐throated Redstart	MYMI	0	1	0
*Myiothlypis coronata*	Russet‐crowned Warbler	BACO	11	7	11
*Myiothlypis nigrocristata*	Black‐crested Warbler	BANI	9	10	11
*Anisognathus igniventris*	Scarlet‐bellied Mountain‐Tanager	ANIG	8	9	7
*Catamblyrhynchus diadema*	Plushcap	CADI	1	1	4
*Catamenia analis*	Band‐tailed Seedeater	CAAN	0	1	0
*Catamenia homochroa*	Paramo Seedeater	CAHO	1	2	0
*Catamenia inornata*	Plain‐colored Seedeater	CAIN	0	11	2
*Conirostrum cinereum*	Cinereous Conebill	COCI	0	10	1
*Conirostrum sitticolor*	Blue‐backed Conebill	COSI	1	0	0
*Diglossa cyanea*	Masked Flowerpiercer	DICY	11	11	11
*Diglossa humeralis*	Black Flowerpiercer	DIHU	10	11	11
*Dubusia taeniata*	Buff‐breasted Mountain‐Tanager	DUTA	0	9	5
*Tangara vassorii*	Blue‐and‐black Tanager	TAVA	9	5	7
*Thlypopsis ornata*	Rufous‐Chested Tanager	THOR	2	5	5
*Thlypopsis superciliaris*	Superciliaried Hemispingus	HESU	7	6	7
*Arremon assimilis*	Gray‐browed Brushfinch	BUTO	10	5	11
*Atlapetes latinuchus*	Yellow‐breasted Brushfinch	ATLA	8	10	11
*Zonotrichia capensis*	Rufous‐collared Sparrow	ZOCA	0	11	2
*Amblycercus holosericeus*	Yellow‐billed Cacique	AMHO	4	1	7
*Spinus magellanicus*	Hooded Siskin	CAMA	0	2	0

Note

Data presented include the number of annual occurrences of each species in each habitat from 2006 to 2016.

**FIGURE 3:**
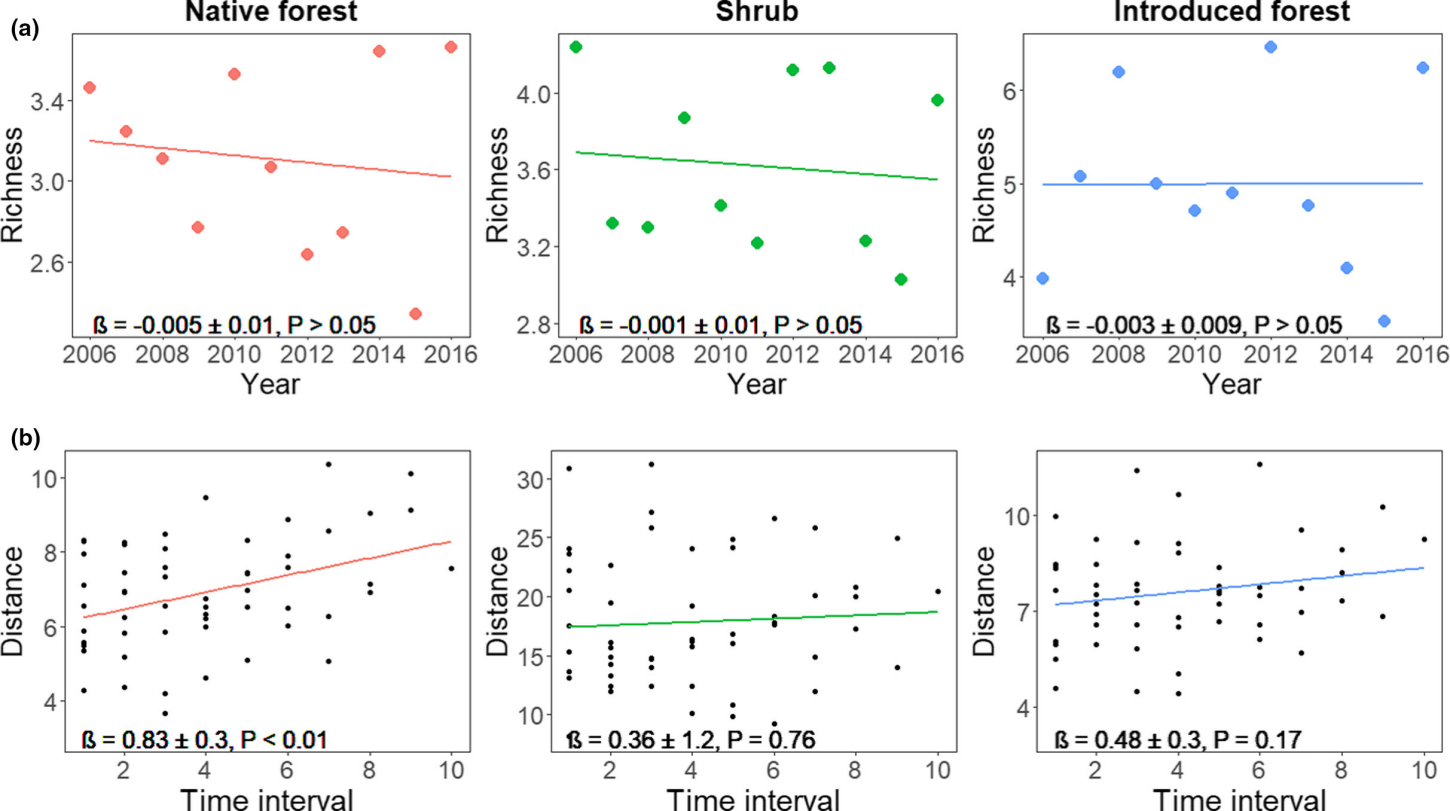
(a) Temporal change in species richness in native forest, shrub, and introduced forest habitats located in Cajas National Park and Mazán Reserve in the highlands of southern Ecuador. Richness is expressed as mean annual richness in 100 mist nets hours. (b) Time‐lag analysis of temporal rate of change in community composition in each habitat. Distance is the Euclidean distance of community composition at increasing time lags among sampling periods. In time‐lag analysis, steeper slopes indicate greater community composition change

Observed annual species richness did not change in any of the habitats over time (Figure 3a). These results are not influenced by sampling artifacts as the detectability of species richness has been constant over time (native forest *F*_1,19_ = 0.62, *p*‐value = 0.45; native shrubs *F*_1,19_ = 0.52, *p*‐value = 0.48; introduced forest *F*_1,19_ < 0.01, *p*‐value = 0.93).

The time‐lag analysis revealed a significant temporal rate of change in community composition only in the native forest (Figure 3b); the other two habitats did not present a temporal trend (Figure 3b). The same patterns of temporal community composition change in the native forest, and stability in the native shrubs and the introduced forest, did not differ when data of dominant species were used (Figure S1).

**FIGURE 4:**
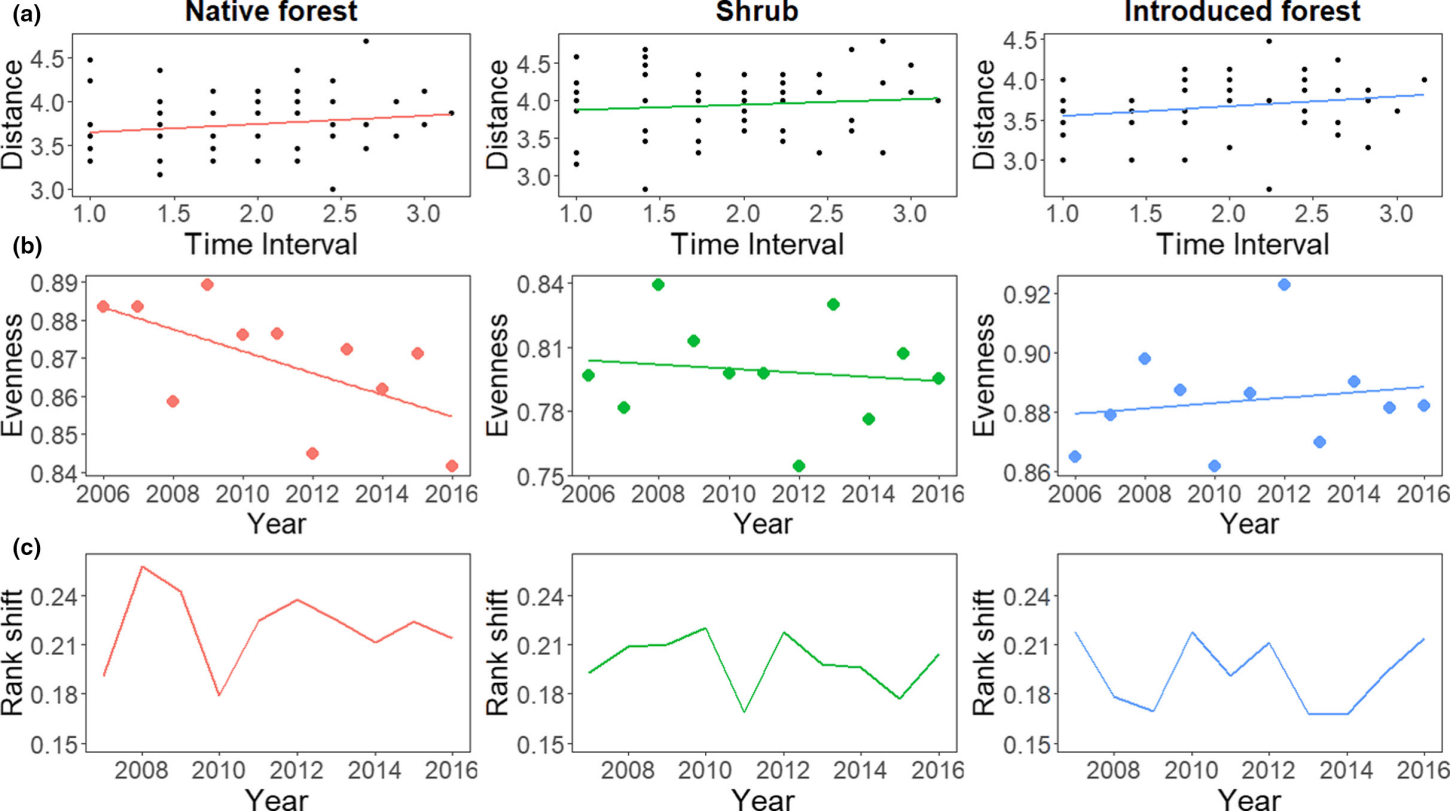
(a) Time‐lag analysis between time periods measuring the rate of taxonomic turnover using only presence/absence data in native forest, shrub, and introduced forest habitats located in Cajas National Park and Mazán Reserve in the highlands of southern Ecuador. (b) Temporal change in species evenness measured using Pielou's index. (c). Mean rank shifts in species abundances across time in each habitat

There was no temporal trend in the rate of taxonomic turnover in any of the habitats (Figure 4a, Table 2). Moreover, species evenness declined in the native forest, but not in the other habitats (Figure 4b, Table 2). Mean rank change was variable across habitats, without any particular temporal trend (Figure 4c).

**TABLE 2:** Result of statistical models applied to data about assemblages of birds captured with mist nets in native forest, native shrub, and exotic forest habitats in Cajas National Park and Mazán reserve, southern highlands of Ecuador

Model	Habitat	Coefficient (95% CI)	*R* ^2^	*p* value
a) Taxonomic turnover	Native forest	0.09 (−0.06, 0.25)	0.02	0.22
	Native shrub	0.07 (−0.12, 0.26)	0.01	0.45
	Introduced forest	0.12 (−0.02, 0.27)	0.04	0.11
b) Evenness	Native forest	−0.02 (−0.01, −0.05)	0.37	0.04
	Native shrub	−0.01 (−0.0, 0.03)	0.01	0.60
	Introduced forest	0.00 (−0.02, 0.04)	0.03	0.60

Note

(a) Time‐lag analysis of the rate of taxonomic turnover over time evaluated with linear regressions. (b) Species evenness over time evaluated with linear mixed models.

**FIGURE 5:**
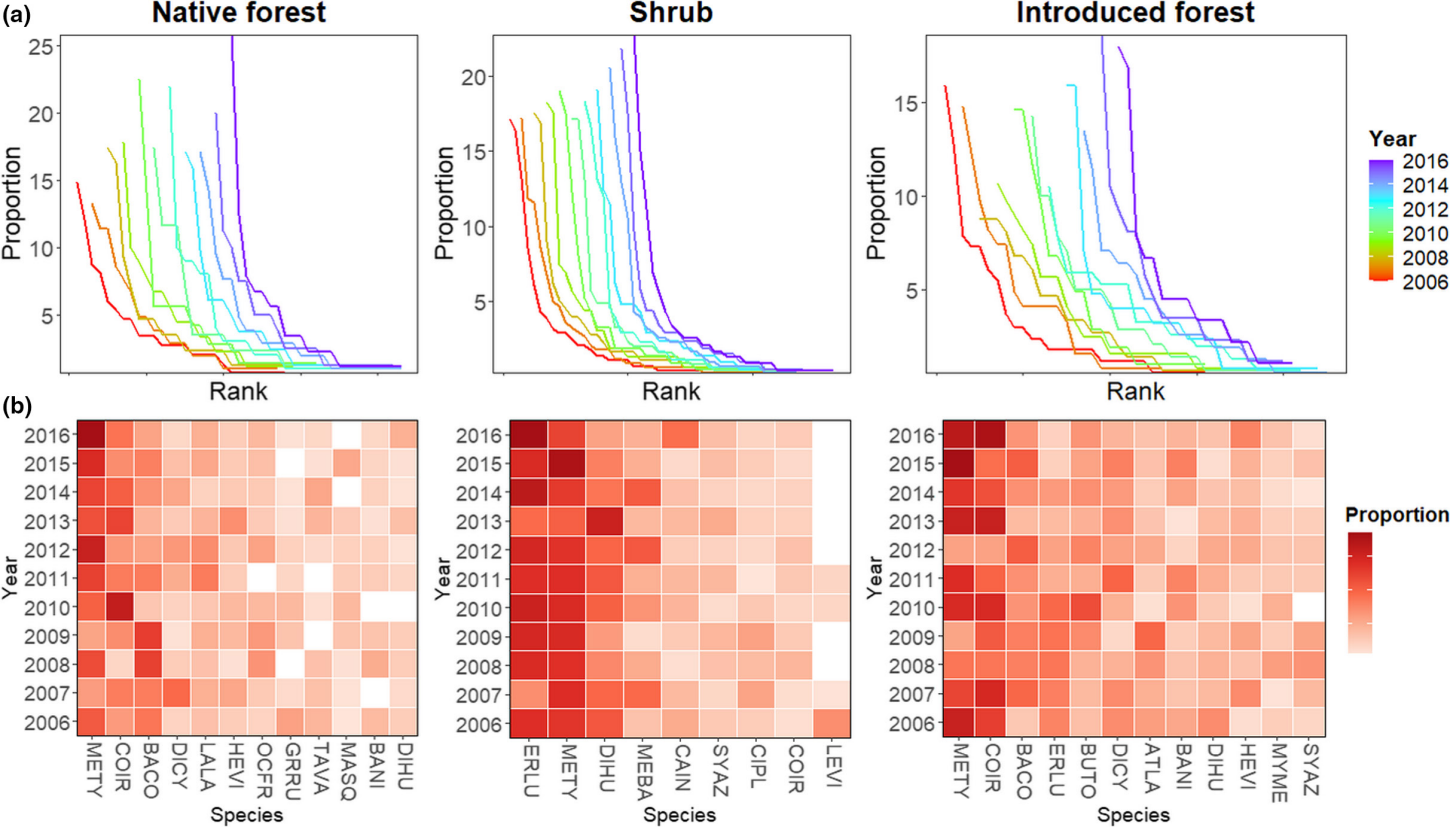
(a) Annual rank abundance distribution plots of the bird assemblage in native forest, shrub, and introduced forest from 2006 to 2016 in Cajas National Park and Mazán Reserve in the highlands of southern Ecuador. The *y*‐axis represents the proportion of abundances of each species, while the x‐axis represents the rank of each species. Each color line represents a different year, and within years, species are ordered from the species with the highest rank to the species with the lowest rank. (b) Heat maps depict the proportions of the dominant species within assemblages from 2006 to 2016 in each habitat. The y‐axis represents the proportion of abundances of each species, while the x‐axis represents species names. Codes of species names correspond to complete names presented in Table 1

Rank abundance distribution plots over time in each habitat showed that in native forest and native shrub habitats, the proportional abundance of the highest ranked species had increased from 2006 to 2016 (Figure 5a). Heat maps, based on proportional abundances of dominant species of assemblages over time in each habitat (Figure 5b), showed an increase in dominance of top‐ranked species in the native forest. In particular, *Metallura tyrianthina* and *Coeligena iris* increased in abundance over time relative to other species, while *Basileuterus coronatus* decreased in dominance. In the native shrub habitat, *Eriocnemis luciani* increased in dominance over time.

## 4 DISCUSSION

Using a unique dataset from the tropical Andes, we examined how species richness and community composition of birds changed over 11 years in three habitats located in high‐elevation protected areas. Taxonomic richness remained constant in native forest, shrub, and introduced forest habitats. However, we detected taxonomic reordering in the native forest, where evenness of the distribution of species decreased over the studied period. This trend, resulting in increased dominance of some species, while others became rare in the native forest, is of conservation concern because it indicates that biodiversity could be changing over time even in the absence of local disturbances. Changes in avian diversity in the absence of local disturbances have been found previously in other tropical ecosystems (Blake & Loiselle, 2016; Escobar et al., 2008; Sigel et al., 2006; Stouffer et al., 2021). The continuation of our monitoring efforts will reveal if these changes in evenness will result in local extinctions or other detrimental losses. Our findings in the native forest correspond to those of several other studies where species richness remained constant over time while changes were recorded in the identity of species in the avian community, and/or their abundances (Blowes et al., 2019; Thibault et al., 2004).

Constant species richness over time across all habitats agrees with global analyses that suggest that at the local scale species richness is relatively stable (Blowes et al., 2019; Dornelas et al., 2014; Thibault et al., 2004). However, these results contrast with those of a previous study in the Mazán Valley which was based on two temporal samples, 1994–1995 and our first sampling period in 2006–2007. In that analysis, richness appeared to decline in native and introduced forests (Latta et al., 2011). This difference may be the result of stochastic variation of communities that were sampled at two different points in time (Fournier et al., 2019), or differences in sampling protocols as compared to the present study. Alternatively, given extensive deforestation in the region (White & Maldonado, 1991), regionally rare species may have already been absent when we started sampling, and this may have affected estimated species richness. This possibility is supported by Latta et al. (2011) who found that those species present in 1994–1995 and absent 11 years later were considered to be rare in Cajas National Park. *Hemispingus verticalis* and *Cnemathraupis eximia*, for example, are among those rare species that were detected in the 1994–1995 study period, but have not been recorded since we started sampling in the native forest in 2006. Therefore, since 2006, we might be monitoring an already impoverished avian community already suffering from the loss of species. This same factor could explain the stability in taxonomic turnover we detected in all habitats, where avian communities in the region are currently composed of multiple‐disturbance‐tolerant species that are able to maintain populations over time in different habitat types (Tinoco et al., 2019).

Our time‐lag analysis showed that species composition is becoming more dissimilar over time in the native forest. This change in the native forest is likely determined by taxonomic reordering of the community. Although the decline in evenness over time was marginally significant, our results suggest that some species became more dominant over time in this habitat. We expected that the most undisturbed site would have the lowest rates of change in community composition because the internal dynamics of undisturbed forests should be governed by stochastic demographic variation in populations (Jackson & Sax, 2010; Stegen et al., 2013). This contrasts with populations in disturbed habitats that are often transient, leading to high levels of temporal community composition change (White et al., 2010). For instance, across habitats comparisons have shown that temporal composition change is higher in disturbed or successional environments compared to undisturbed habitats (Kampichler et al., 2014; Stegen et al., 2013).

Several interacting environmental factors may have influenced the change in evenness in the native forest, including successional processes in the local vegetation, regional land‐use change, and global climate change. Detailed measures of forest structure in the native forest showed little variation in canopy cover, and number of trees and shrubs between 2007 and 2013 (Tinoco et al., 2019), suggesting that local vegetation dynamics have not strongly influenced observed changes in assemblage evenness. As local assemblages are highly connected to dynamics of the regional species pool, it is possible that changes in the native forest are a result of land‐use change operating beyond the boundaries of the Mazán reserve (Latta et al., 2011). This is difficult to evaluate as there are no updated fine‐scale estimates of land‐use change in the region. Transformation of mountain forest to pastures is an ongoing process in the Ecuadorian Andes (Ministerio del Ambiente, 2016), and habitat conversions to promote grazing continue to alter the composition of bird assemblages (Palacio et al., 2020; Santillán et al., 2019; Tinoco et al., 2018). Finally, tropical mountains are highly sensitive to climate change (Urrutia & Vuille, 2009), and recent evidence suggests range shifts and even extirpations of birds in the tropical Andes as a result of climate change impacts (Feeley et al., 2011; Freeman et al., 2018). Detailed regional information on land conversion rates and climate change, combined with broader monitoring of species composition across space, is required to better explore the driving factors of biodiversity change at local scales as seen here.

Temporal changes in evenness signal an assemblage that is undergoing a transitional phase (Smith et al., 2009), usually triggered by a disturbance in the environment (Dornelas, 2010; Kim et al., 2013; Matthews & Whittaker, 2015; Shurin, 2007). If evenness continues to decline in the native forest, it is possible that local extinctions might occur and the assemblage may reach a new species equilibrium with only a subset of the original species (Jackson & Sax, 2010; Smith et al., 2009; Supp & Ernest, 2014). However, species reordering can be complex and nonlinear, as it can be the result of multiple ecological factors that operate at different scales with varying intensities (e.g., climate change, land‐use change, species interactions) (Collins et al., 2020). The continued monitoring of populations in the study area will provide value information about the future dynamics of bird assemblages and show if the observed temporal decrease in evenness ultimately results in local extinctions.

Exploring temporal changes in biodiversity across different habitats in the tropical Andes is necessary for predicting the long‐term consequences of global environmental change, as this region harbors a large proportion global diversity (Laurance et al., 2012). Because of a lack of habitat‐level replicates, our study cannot be used to quantitatively compare rates of community change among habitats; we acknowledge that limitation. Yet, it is striking that the less altered habitat, native forest, has a higher rate of composition change than the more altered shrub and introduced forest habitats. Moreover, the native forest in Mazán reserve is managed under strict protection practices; thus, our results suggest that well‐protected reserves are not immune from the current global change. Temporal declines in richness, and changes in community composition, have also been observed in other strictly protected areas of the tropical region, bringing into question whether protected areas are sufficient for maintaining biological diversity in the long term (Laurance et al., 2012; Malhi et al., 2014; Stouffer et al., 2021). Unfortunately, there are very few studies documenting temporal trends in Andean biodiversity, even in well‐studied groups such as birds. Our study highlights the value of long‐term studies in the tropical Andes, as we show that species composition of birds in a montane forest is changing, providing additional support for global trends in biodiversity change (Blowes et al., 2019; Dornelas et al., 2014; Newbold et al., 2020).

## Supporting information

Fig S1

## Data Availability

The data that support the findings of this study are openly available in the Dryad Digital Repository: https://doi.org/10.5061/dryad.r2280gbds (Tinoco et al., 2021).
