# Supplementary material for: Temporal stability in species richness but reordering in species abundances within avian assemblages of a tropical Andes conservation hot spot
Source: Biotropica. 2021 Oct 5;53(6):1673–84. doi: 10.1111/btp.13016 (PMC9293307; doi:10.1111/btp.13016)

SUPPORTING INFORMATION

**Temporal stability in species richness but reordering in species abundances within avian assemblages of a Tropical Andes conservation hot spot**

Boris A. Tinoco^1^, Steven C. Latta^2^, Pedro X. Astudillo^1^, Andrea Nieto^1^, Catherine H. Graham^3^*.

^1^ Escuela de Biología, Universidad del Azuay, Cuenca, Ecuador

^2^ National Aviary, Allegheny Commons West, Pittsburgh, Pennsylvania, USA

^3^Swiss Federal Research Institute WSL, Birmensdorf, Zurich, Switzerland

*Corresponding author

Catherine H. Graham. Email: Catherine.graham@wsl.ch

Figure S1. Time lag analysis of temporal rate of community change using data of dominant species in native forest, shrub and introduced forest habitats located Cajas National Park, and Mazan Reserve in the highlands of southern Ecuador. Distance is the Euclidean distance of community composition at increasing time lags among sampling periods. Dominant species were defined as the minimum number of species that together represent 50% of all individuals that are part of the assemblage. The annual number of species that fell under the dominant category across the study period varied between 4 to 6 species in the native forest, 4 to 6 species in the shrubs, and 5 to 8 species for the introduced forest.


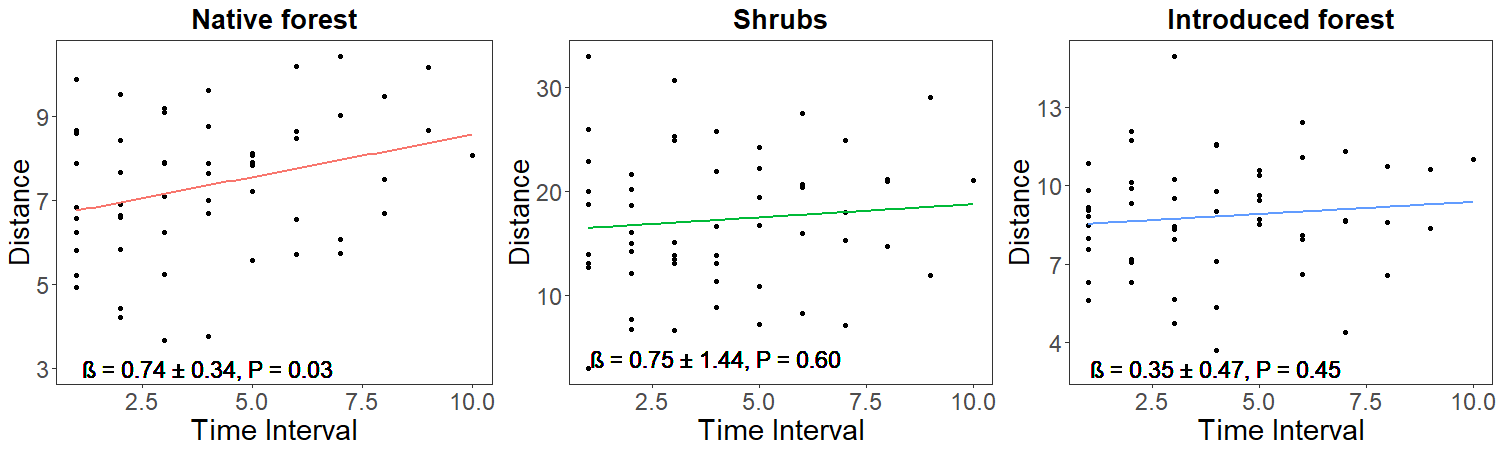

Supplement: Supplementary file 1 — Fig S1 [file BTP-53-1673-s001.docx]
